# Preclinical Evaluation of a Zinc Oxide Nanoparticle–Loaded Amniotic Membrane as a Bioactive Scaffold for Partial‐Thickness Burn Healing in Rats

**DOI:** 10.1155/bmri/7362331

**Published:** 2026-04-23

**Authors:** Seyedeh-Sara Hashemi, Ali-Akbar Mohammadi, Seyed Behzad Jalali, Seyedarad Mosalamiaghili, Alireza Rafati, Mojtaba Ghaedi

**Affiliations:** ^1^ Burn and Wound Healing Research Center, Shiraz University of Medical Sciences, Shiraz, Iran, sums.ac.ir; ^2^ Department of Comparative Biomedical Sciences, School of Advanced Medical Sciences and Technologies, Shiraz University of Medical Sciences, Shiraz, Iran, sums.ac.ir; ^3^ Division of Plastic and Reconstructive Surgery, Department of Surgery, Shiraz University of Medical Sciences, Shiraz, Iran, sums.ac.ir; ^4^ Student Research Committee, Shiraz University of Medical Sciences, Shiraz, Iran, sums.ac.ir; ^5^ Department of Food Science and Technology and Nutrition, Islamic Azad University, Sarvestan, Sarv.C, Sarvestan, Iran, tiau.ac.ir; ^6^ Department of Surgery, School of Medicine, Jahrom University of Medical Sciences, Jahrom, Iran, jums.ac.ir

**Keywords:** burn wound, human amniotic membrane, wound healing, zinc oxide

## Abstract

Burn wound infections continue to be a leading cause of morbidity and mortality despite advancements in medical treatments. Human amniotic membrane and zinc oxide nanoparticles have individually shown promise in enhancing wound healing. This study evaluated the efficacy of a novel composite scaffold made of zinc oxide nanoparticles–impregnated human amniotic membrane for treating second‐degree burn wounds. Human amniotic membrane was isolated from cesarean‐delivered placentas and loaded with nanoparticles via ultrasonic dispersion. The composite scaffold was characterized using scanning electron microscopy and energy‐dispersive x‐ray spectroscopy, which confirmed uniform nanoparticle distribution. Human fibroblast cell culture studies demonstrated enhanced cell viability and adhesion in the composite scaffold compared with controls. In a rat model of second‐degree burns, the nZnO‐impregnated human amniotic membrane significantly improved wound healing, evidenced by faster reepithelialization, reduced inflammation, and complete tissue regeneration by Day 14. These findings suggest that the combination of nZnO and human amniotic membrane offers superior wound healing properties over traditional treatments, with potential applications for burn care. Future studies should explore human trials to further validate the clinical potential of this novel approach.

## 1. Introduction

Rapid and effective wound healing is essential for burn injury patients [1]. Although the mortality rate has decreased with advancements in medical techniques such as fluid resuscitation, nutritional support, early excision of wound eschar, and skin grafting [2, 3], infection and sepsis remain prevalent and are often fatal. Approximately 51%–75% of patients with major burns succumb to these complications [4]. Infections, in particular, are a major obstacle to the healing process and are the leading cause of mortality [5]. Burn wound infections can lead to delayed healing, increased pain, prolonged hospital stays, and higher healthcare costs [6]. The emergence of multidrug‐resistant (MDR) bacteria has further complicated the treatment of these infections [7]. Managing antibiotic‐resistant pathogens has become a significant challenge for clinicians, prompting researchers to explore innovative approaches for preventing burn wound infections and improving healing outcomes in recent years [8, 9].

Human amniotic membrane (hAM), obtained from the placenta, has attracted considerable attention for its role in wound healing due to its unique properties [10, 11]. The structure of hAM resembles that of human skin, as it is derived from the ectoderm, making it effective in mitigating water and heat loss from the wound while preventing bacterial contamination [12]. Furthermore, hAM is superior to other allograft and xenograft options as a biological dressing, as it alleviates pain, promotes reepithelialization, and reduces heat, protein, and energy loss [13]. The availability and low cost of hAM make it an exceptional choice for wound dressings, especially in economically disadvantaged regions [14, 15].

In recent years, an increasing body of research has focused on the efficacy of various dressings, including amniotic membrane, as well as different antimicrobial agents to prevent wound infections and enhance healing outcomes [16–18]. Among these antimicrobial agents, zinc oxide nanoparticles (nZnO) have shown promising results in promoting wound healing [19–21]. nZnO exhibits excellent antimicrobial activity [17, 22] against a wide range of pathogens, including antibiotic‐resistant strains. Additionally, they possess anti‐inflammatory properties and can stimulate tissue repair processes [23, 24]. In contrast to various antimicrobial substances like gold, silver, or titanium dioxide, nZnO exhibits reduced toxicity [25] and serves as a source of zinc (Zn), a vital element required for the proper metabolism, immune system functionality, and facilitation of wound healing processes in the human body [26, 27].

The primary objective of this study is to compare the efficacy and clinical outcomes of amniotic membrane dressings and nZnO‐impregnated hAM dressings in the treatment of second‐degree burn wounds. This research is aimed at contributing to the existing body of knowledge and provide healthcare professionals with valuable insights to guide the selection of appropriate dressings for second‐degree burn wounds.

## 2. Methods

### 2.1. Ethical Approval

The study received ethical approval from the “Ethical Review Committee” of Shiraz University of Medical Sciences, Shiraz, Iran [*omitted due to anonymity*]. All procedures involving human and animal subjects adhered to the principles outlined in the Helsinki Declaration. hAM and circumcision skin were obtained following informed consent, and all necessary serological tests for HIV, hepatitis B and C, and syphilis were conducted, yielding negative results. Animal handling and dissection were performed in accordance with the ARRIVE guidelines. No placental tissue was sourced from prisoners. Written informed consent was obtained from all patients enrolled in the study, and all methods were conducted following relevant ethical guidelines and regulations.

### 2.2. Construction of hAM

The hAM used in this study was obtained immediately after a cesarean section, following informed consent. Serology tests for HIV, hepatitis B and C, as well as syphilis, were conducted on the donor, all yielding negative results. The hAM was washed using sterile normal saline to remove blood clots, using a sterile cotton swab. The inner amniotic membrane was then separated from the remaining parts of the chorion through blunt dissection. The membrane was placed on a wide nitrocellulose membrane with the epithelial surface facing upwards, following procedures described in previous studies [11, 28].

Pieces of hAM measuring 5 × 5 cm were isolated, and each piece was submerged in phosphate‐buffered saline (PBS) until nZnO were loaded onto them using an ultrasonic bath. The amniotic membrane was immersed in a suspension of nZnO, and ultrasonic waves were applied to the suspension using an ultrasonic bath. The cavitation process generated tiny bubbles that imploded, releasing energy, which dispersed and evenly distributed the nZnO across the membrane. Microbiological tests were performed on all original and prepared membranes, as well as the fluids used in the process.

### 2.3. Evaluation of the Amnion With Nanoparticles

The morphological and structural characteristics of the Amnio–ZnO nanoparticles were examined using a combination of electron microscopy and surface‐charge analysis. Briefly, scanning electron microscopy (SEM) was employed at a voltage of 20 kV to assess surface topology and overall particle morphology (SEM, TESCAN VEGA 3, Czech Republic), whereas transmission electron microscopy (TEM) provided high‐resolution visualization of particle size, shape, and internal structure (JOEL‐JEM2100). Before imaging, samples were sputter‐coated with a thin layer of gold using a sputter coater (Q150R‐ES, Quorum Technologies, United Kingdom). Elemental analysis for Zn, carbon (C), nitrogen (N), and oxygen (O) in the scaffold was performed using energy‐dispersive x‐ray (EDX) mapping, enabling verification peaks along with amnion‐derived organic components. The porosity of the scaffold was measured by image processing with ImageJ software (National Institutes of Health, United States) Version 1.53. Finally, to evaluate colloidal behavior, the zeta potential of the nanoparticle suspension was measured by electrophoretic light scattering at 25°C in aqueous medium (Horiba SZ‐100).

### 2.4. Fibroblast Cell Culture

Fibroblast cells were cultured from skin obtained through circumcision. The skin was washed with PBS containing penicillin–streptomycin and the hypodermis was removed. The skin was then cut into 1 × 1 − cm pieces, placed in a dispase solution, and refrigerated for 18 h to facilitate the separation of the epidermis from the dermis. After this period, the dispase solution was removed, and the skin pieces were incubated in trypsin to isolate fibroblast cells from the dermis. These cells were then transferred to a culture flask and incubated at 37°C in a CO2 incubator. The cells were allowed to adhere to the bottom of the flask, and their growth was monitored daily using an inverted microscope. All procedures were carried out under sterile conditions in a sterile hood [29].

### 2.5. Flow Cytometry

To confirm the isolated cells as fibroblasts, the cultured cells were trypsinized and separated from the flask′s surface. Mouse antihuman antibodies for CD10 (phycoerythrin, PE), CD34 (peridinin chlorophyll protein, PerCP), CD45 (fluorescein isothiocyanate, FITC), and CD106 (allophycocyanin, APC) were added to the samples and incubated. The cells were analyzed using a fluorescence‐activated cell sorter (FACSCalibur, Becton Dickinson, United States).

### 2.6. Cell Proliferation and Viability Assay Using 3‐(4,5‐Dimethylthiazol‐2‐Yl)‐2,5‐Diphenyltetrazolium Bromide (MTT)

Scaffold punch pieces were placed in a 96‐well plate and 10^4^ fibroblast cells were seeded onto each scaffold. The plates were incubated for 24 and 48 h. After incubation, 50 *μ*L of MTT solution was added to each well, and the plates were incubated for an additional 4 h. After removing the MTT solution, 100 *μ*L of dimethyl sulfoxide (DMSO) was added to each well, and the plates were kept in the dark at room temperature for 30 min. Absorbance was measured at a wavelength of 570 nm using an ELISA reader [30–32].

### 2.7. DAPI (4 ^′^, 6‐Diamidino‐2‐Phenylindole) Staining

DAPI staining was performed after 48 h of incubation by adding 100 *μ*L of Triton X‐100 to each well and incubating for 15 min. The wells were then washed with PBS, and 100 *μ*L of paraformaldehyde was added and incubated for 1 h. A 1:1000 dilution of the DAPI solution was added to each well and incubated for 15 min. After washing with PBS, the wells were photographed using a fluorescence microscope.

### 2.8. Cell Adhesion and Proliferation Measurement

Scaffolds were placed in 12‐well plates, and 30,000 fibroblast cells were added to each scaffold. The plates were incubated for 48 and 72 h. To fix the cells to the scaffolds, a 2.5% glutaraldehyde solution was used. The scaffolds were immersed in the solution and stored in a refrigerator for 4 h. After removal of the glutaraldehyde solution, the scaffolds were rinsed with deionized water and dehydrated using ethanol solutions (25%, 50%, 75%, and 100%) for 20 min each. The scaffolds were allowed to air dry.

### 2.9. Modeling Grade 2 Burns in Rat Animal Model and Therapeutic Interventions

Thirty‐six adult rats weighing 180–200 g were used in this study. The animals were obtained from the Experimental Medical Center and Comparative Medicine, Shiraz University of Medical Sciences. They were kept under standard conditions with a 12‐h light/dark cycle, at a temperature of 22°C ± 5°C and relative humidity of 60*%* ± 5*%*. Each rat was housed in an individual cage and provided with standard laboratory diets and water. A Grade 2 burn wound model was created on the dorsal region of the rats. The animals were randomly divided into three groups (*n*  =  12 per group). Rats were anesthetized with ketamine and xylazine (100 and 5 mg/kg, respectively), and the shaved dorsal skin area was disinfected with 10% povidone–iodine. A circular burn wound (10‐mm diameter) was created by applying 90°C water for a standardized duration under controlled conditions. Skin samples were collected at 3th, 7th, and 14th days postburn for analysis. The groups were as follows:

• Group 1: Treatment with amniotic membrane and nZnO.
• Group 2: Control group with amniotic membrane treatment only.
• Group 3: Negative control group with no treatment.

### 2.10. Macroscopic Evaluations of Burn Wounds

At 3‐, 7‐, 10‐, and 14‐day posttreatment, rats were anesthetized with ether inhalation, and photographs of the burn wounds were taken. The percentage of wound contraction was calculated using ImageJ software with the following formula: Wound contraction (*%*) = [(area on Day 0–area on Day *n*)/area on Day 0] × 100.

### 2.11. Histological and Stereological Evaluations of Full‐Thickness Wounds

At 3rd, 7th, and 14th‐day postburn, rats were euthanized with an overdose of ketamine/xylazine. Tissue samples from the wound site were collected and fixed in 10% formalin. The samples were then dehydrated in ethanol and embedded in paraffin. Serial sections (10 *μ*m thick, spaced 100 *μ*m apart) were made using a rotary microtome [33]. The sections were mounted on glass slides, deparaffinized, and stained with hematoxylin and eosin (H&E) [34]. The stained tissue slides were analyzed using a light microscope (Olympus CX41, Japan) by a blinded observer [35].

### 2.12. Statistical Analysis

Data were analyzed using the Statistical Package for Social Sciences (SPSS) Version 26 (IBM, United States). Descriptive statistics, including percentages, frequencies, means, medians, and standard deviations, were calculated where applicable [32]. The normality of the data was assessed using the Shapiro–Wilk test. One‐way ANOVA was used to compare the efficacy of the different groups with the Tukey post hoc test [36]. *p* < 0.05 was considered significant difference [37].

## 3. Results

### 3.1. SEM and TEM

Electron microscopy of the decellularized scaffold revealed a highly porous structure, ensuring effective decellularization with no residual cells. The uniform pore size within the amnion scaffold provides an ideal environment for cell attachment and nutrient transport, indicating its suitability for tissue engineering applications (Figure 1).

Figure 1TEM and SEM. Scanning electron microscopy images of human amniotic membrane scaffold treated with zinc oxide nanoparticles. *(a)* Surface morphology of decellularized amnion scaffold without cells, demonstrating a highly porous structure supporting cell attachment and nutrient flow. *(b)* Cross‐sectional view of the scaffold, highlighting its uniform thickness and enhanced porosity, essential for cellular infiltration and nutrient exchange.

**Figure:**
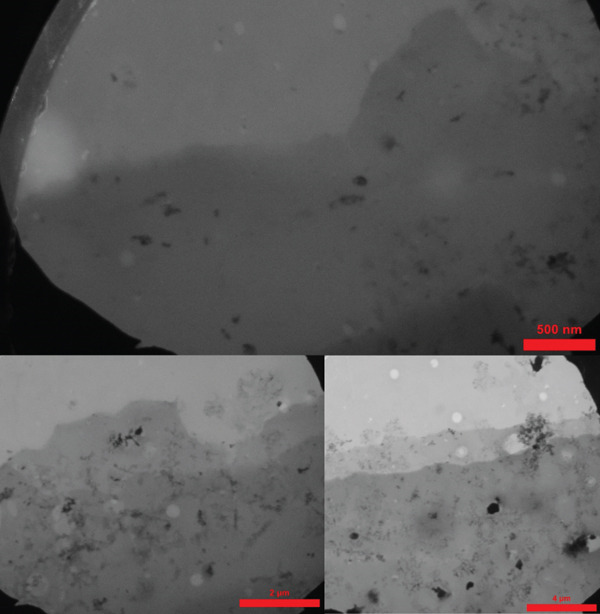
(a)

**Figure:**
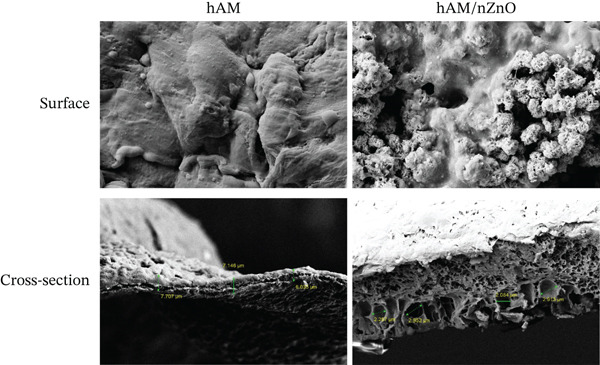
(b)

### 3.2. EDX Mapping

**Figure 2:**
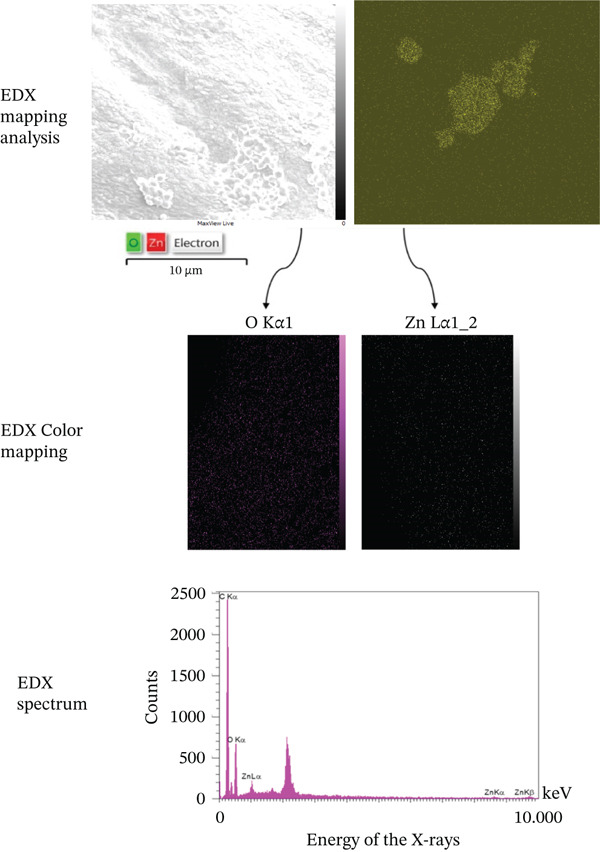
Energy‐dispersive x‐ray spectroscopy (EDX) mapping illustrates the even distribution of zinc oxide nanoparticles across the scaffold′s surface, providing a uniform environment for cell proliferation and attachment.

Figure 2 illustrates the EDX mapping analysis, which confirms the even distribution of nZnO throughout the scaffold. This uniform nanoparticle dispersion enhances the scaffold′s surface properties, facilitating cell adhesion and proliferation by providing consistent biochemical cues across the material.

### 3.3. Zeta Potential

**Figure 3:**
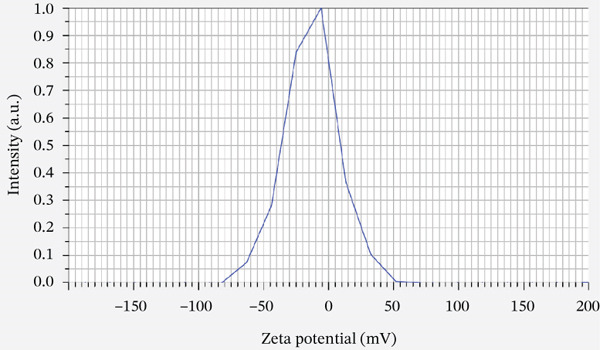
Zeta potential of Amnio ZnO at 25.1°C with dispersion medium viscosity: 0.893 mPa·s, conductivity: 18.199 mS/cm, and electrode voltage: 1.3 V.

The zeta potential of Amnio–ZnO nanoparticles was measured by electrophoretic light scattering at 25°C in aqueous medium with viscosity 0.893 mPa·s, conductivity 18.199 mS/cm, applied electrode voltage 1.3 V. Amnio–ZnO nanoparticles exhibited a mean zeta potential of −13.2 mV, indicating moderate colloidal stability and mean electrophoretic mobility of −0.000103 cm2/Vs (Figure 3).

### 3.4. Identification of Fibroblast Cells

The results of culturing fibroblast cells isolated from human dermal tissue initially showed a spherical shape (Figure 4a). After 24 h of culture, the cells exhibited a spindle‐shaped morphology and adhered to the flask bottom (Figure 4b). Approximately 3–4 days after culturing, a monolayer of cells with a density of 80%–90% was formed on the flask bottom, as shown in (Figure 4c).

Figure 4Morphological characteristics and identification of fibroblast cells cultured on the scaffold. *(a)* Initial spherical morphology of fibroblast cells isolated from human dermal tissue. *(b)* Spindle‐shaped morphology of fibroblast cells after 24 h in culture, adhering to the flask surface. *(c)* Monolayer formation on the flask bottom after 3–4 days of culture, indicating 80%–90% confluency. *(d)* Flow cytometry results for fibroblast cell markers, showing expression of CD10 and absence of hematopoietic and mesenchymal stem cell markers (CD34, CD45, and CD106). CD: cluster of differentiation.

**Figure:**
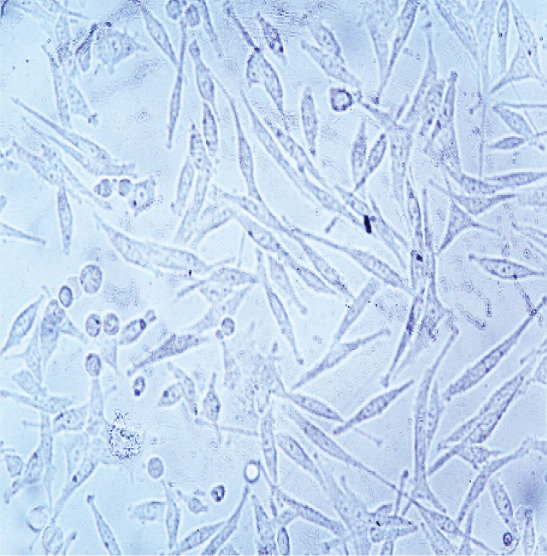
(a)

**Figure:**
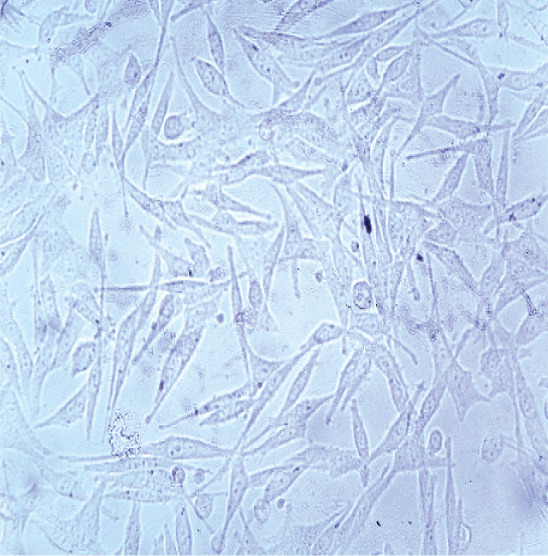
(b)

**Figure:**
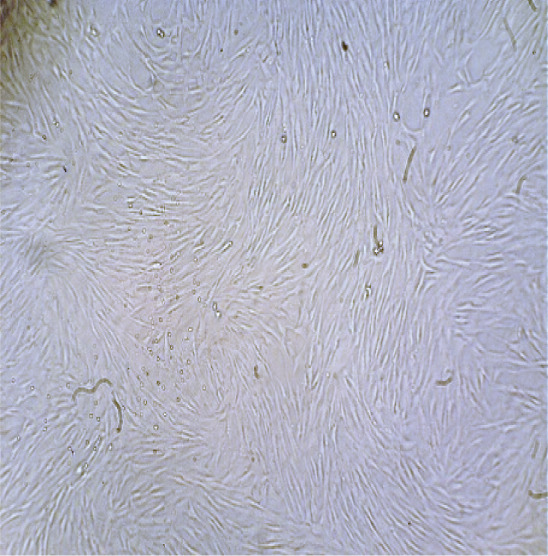
(c)

**Figure:**
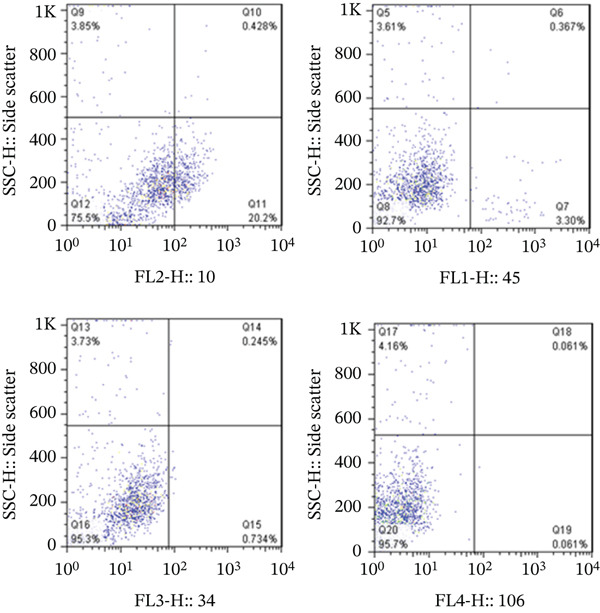
(d)

To confirm the nature of the fibroblast cells isolated from human dermal tissue, flow cytometry analysis was performed. The results indicated that these cells did not express hematopoietic markers, including CD34 and CD45. They also did not express CD106, which is specifically expressed in mesenchymal stem cells and not in fibroblasts. However, they expressed CD10, which is expressed in both mesenchymal stem cells and fibroblasts, but with a higher intensity in fibroblast cells. Therefore, it was confirmed that the cells we used were fibroblast cells. (Figure 4d).

### 3.5. MTT Assay

**Figure 5:**
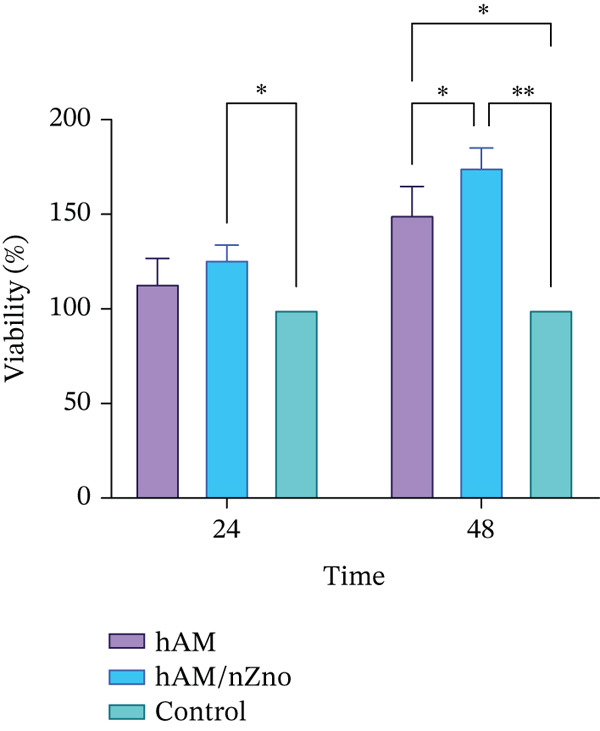
Methyl thiazolyl tetrazolium (MTT) assay results for cell viability and proliferation on the amnion scaffold and amnion with zinc oxide nanoparticles. (a) 24‐h MTT assay results, showing a statistically significant increase in cell viability in the amnion with nanoparticles compared with the control group (*p* < 0.01). (b) 48‐h MTT assay results, demonstrating significantly higher cell viability in both the amnion and amnion with nanoparticles compared with the control (*p* < 0.01 for amnion, *p* < 0.001 for nanoparticles). Amnion with nanoparticles also showed significant improvement over the amnion alone (*p* < 0.05).

The mean cell viability and proliferation percentages based on the MTT assay at 24 h showed a statistically significant increase in the amnion group with nZnO compared with both the amnion‐only group and the control group (*p* < 0.01) (Figure 5).

The mean cell viability and proliferation percentages based on the MTT assay at 48 h showed a statistically significant increase in the amnion group compared with the control group (*p* < 0.01). Additionally, the mean cell viability and proliferation percentages based on the MTT assay at 48 h showed a statistically significant increase in the amnion group with nZnO compared with the control group (*p* < 0.001). Furthermore, the mean cell viability and proliferation percentages based on the MTT assay at 48 h showed a statistically significant increase in the amnion group with nZnO compared with the amnion group (*p* < 0.05) (Figure 5).

### 3.6. Cell Adhesion and Proliferation Measurement

**Figure 6:**
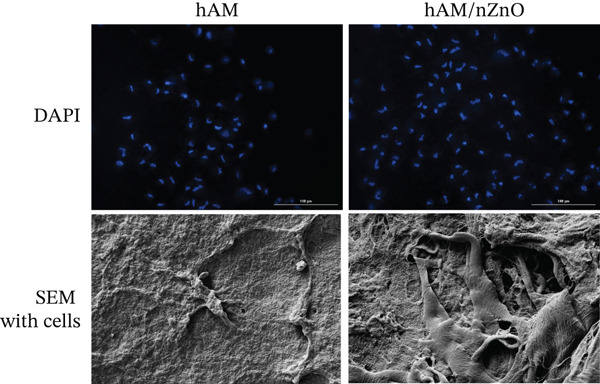
SEM and DAPI staining images of cell adhesion and proliferation on amnion scaffolds. *(a)* SEM images showing fibroblast cell penetration within the amnion scaffold due to its three‐dimensional structure and zinc oxide nanoparticle presence. *(b)* DAPI staining of the amnion with nanoparticles, showing increased cell survival and attachment compared with control. *(c)* SEM images of the scaffold surface, demonstrating a uniform nanoparticle distribution, enhancing cell migration and adhesion. SEM: scanning electron microscopy; DAPI: 4 ^′^,6‐diamidino‐2‐phenylindole.

Electron microscopic images and DAPI staining of the amnion and amnion with nZnO‐treated scaffolds showed that due to the three‐dimensional nature of the electrospun scaffolds and the presence of nZnO, fibroblast cells penetrated and proliferated within the scaffolds, resulting in higher cell survival compared with the hAM groups (Figure 6).

Electron microscopic images of the amnion and amnion with nZnO‐treated scaffolds show a uniform surface of the scaffolds and the homogeneous distribution of nanoparticles on the scaffolds. Additionally, electron microscopic images of the scaffolds with cell seeding indicate the presence of cells and their migration onto the scaffolds (Figure 6).

### 3.7. Macroscopic Evaluations of Burn Wounds

**Figure 7:**
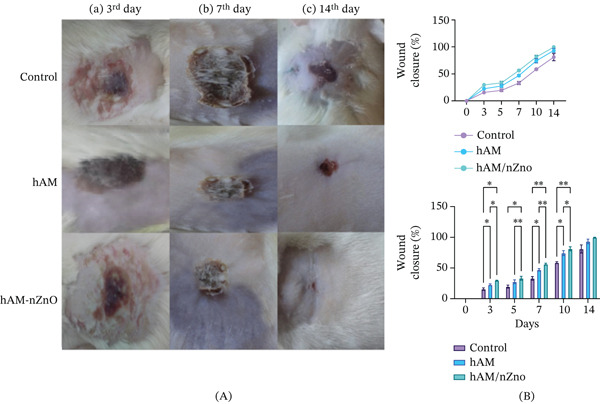
(A) NNN macroscopic images of burn wounds treated with hAM scaffold with and without nZnO over time. (B) wound closure percent in hAM, hAM/nZno, and control groups. Data are expressed as mean ± SD.  ^∗^ *p* < 0.05, and  ^∗∗∗∗^ *p* < 0.001. hAM, human amnion membrane; nZnO, zinc oxide nanoparticles.

Macroscopic changes and wound in animal wounds and closure percentage at the 3, 7, and 14 days after experimental burn injury are displayed in Figure 7. The results indicated that on Days 3 (*p* < 0.05) and 7 (*p* < 0.001) the percent of wound closure was higher in nZnO group than in hAM and control group. Also, the control group exhibits higher inflammation levels than both the amnion and the amnion with nZnO groups, with visible redness and swelling in untreated areas. Day 3, illustrated heightened inflammation in the control group with visible redness and swelling, whereas treated groups showed reduced inflammation. By Day 7, there was a continued reduction in inflammation in treated groups, particularly the amnion with nanoparticles group. By Day 14, the wound showed complete healing in the amnion with nanoparticles group, with no visible signs of inflammation or infection. This result suggests that the scaffold treatments, particularly the nZnO group, help to reduce inflammation in burn wounds, potentially improving healing outcomes.

### 3.8. Pathological Evaluation

**Figure 8:**
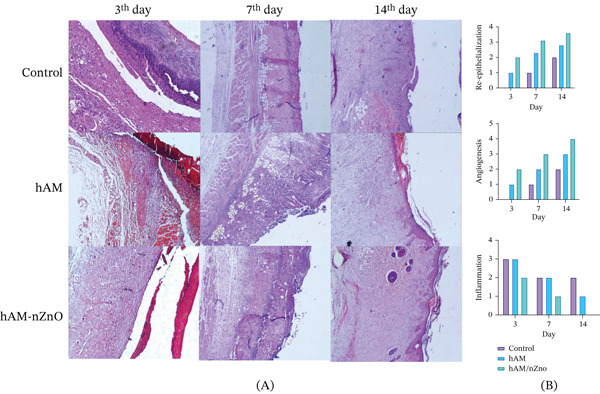
(A) Pathological evaluation of burn wound healing in control, hAM, and ZnO‐hAM groups (H&E staining) and (B) Histopathological scoring of wound healing at 3,7, and 14 days.

Pathological evaluation on Day 7 after burn wound creation and treatment with the amnion scaffold with nZnOs showed a significant effect of this scaffold on the healing process compared with the untreated control group and the amnion‐only group. The control group exhibited coagulative necrosis, complete loss of the epidermis, and absence of tissue regeneration, along with severe vascular dilation and lack of tissue budding. However, in the treatment group, tissue budding and epidermis regeneration had begun (Figure 8).

On Day 14 after treatment, the amnion scaffold with nZnOs showed no inflammation or bleeding in the tissue, and no infection was observed. Incomplete tissue repair with mature tissue budding was observed (Figure 8A). On Day 14, the treatment group showed complete tissue repair with mature tissue budding, neovascularization, and organized collagen fibers. No inflammatory cells or bleeding were observed. However, in the positive control group, tissue budding was still progressing, inflammatory cells were reduced, and tissue organization was observed. The macroscopic and histopathological images reveal faster healing in the amnion with nZnO‐hAM group, showing reduced inflammation, advanced tissue regeneration, and complete repair by Day 14. In contrast, the control group exhibits prolonged inflammation, incomplete epidermal repair, and disorganized collagen. The hAM group shows moderate healing with some remaining inflammation (Figure 8B).

## 4. Discussion

Our study shows that incorporating nZnO into an already healing substance like hAM scaffolds accelerate the healing of second‐degree burn wounds in a rat model and could hopefully be in use for human models. Our findings showed enhanced reepithelialization, reduced inflammation, and increased collagen deposition in the ZnO‐hAM group versus controls. To our knowledge, this is the first study that has provided a burn healing method by combining ZnO nanoparticles with placental tissue.

As the pathogens showing resistance to various antibiotics are growing to cause mortality and morbidity in burn wounds [38], the investigation for finding the cheap and easy way to avoid these harmful infections are becoming increasingly important. The wound healing properties of nZnO nanoparticles are well‐documented in the literature, supporting our findings [39, 40]. The nZnOs are getting more and more popular; and even showing results as effective as colistin [41]. Multiple studies have reported that nZnO exhibit antibacterial activity against various pathogens such as *Staphylococcus aureus*, *Bacillus cereus*, *Salmonella enterica*, and *Klebsiella pneumoniae* standard isolates [42–46], by disrupting cell membranes and inducing oxidative stress [47, 48]. Moreover, nZnO promote key processes involved in tissue regeneration. Wiesmann et al. [49] demonstrated that nZnO stimulates the migration and proliferation of fibroblasts, upregulate collagen production, and support angiogenesis—all critical events in the proliferative phase of healing. Similarly, Raguvaran et al. [50] reported increased viability and growth of fibroblasts seeded on nZnO‐loaded scaffolds. The significant tissue budding and collagen organization seen with our scaffold by Day 21 postburn align with such evidence.

hAM has gained notable attention for its structural compatibility with the corneal basement membrane, leading to its use in ocular surface repair, chronic wound treatment, and broader applications in regenerative medicine. Its versatility has enabled successful applications across various fields, from ocular and dermal healing to tissue engineering [51–55]. But the recent study by Ramasamy et al. [56] shows that the nZnOs could be utilized in addition to hAm for enhanced corneal wound healing and infection prevention. Their study investigates the preparation and development of wound dressings derived from unprocessed hAM dressings significantly enhances their therapeutic efficacy for corneal wound healing and infection control. The nZnO‐hAM displayed a broad‐spectrum antibacterial effect, effectively inhibiting biofilm formation in both Gram‐positive bacteria, such as *S. aureus*, *Streptococcus mutans*, *Enterococcus faecalis*, and *Leptotrichia fusiformis*, and Gram‐negative bacteria, including *Shigella sonnei*, *Pseudomonas aeruginosa*, *Proteus vulgaris*, and *Citrobacter freundii*.

Fibroblast cells, play a crucial role in extracellular matrix synthesis and tissue repair [57], served as the cornerstone of our investigation. The morphological transition observed in cultured fibroblast cells further stressed their readiness for regenerative tasks. Initially, these cells exhibited a spherical shape, a characteristic consistent with their quiescent state [58, 59]. However, within 24 h of culture, they underwent a transformation, adopting a spindle‐shaped morphology and firmly adhering to the flask bottom. This morphological shift signifies their activation and adaptation to the scaffold environment [60]. Khorasani et al. [61] explored the interaction between nZnO and fibroblast cells, specifically in mouse fibroblast cells (L‐929), in the context of potential wound dressing applications. Their results from cell viability tests indicated that all scaffold samples, including those containing nZnO, showed no toxicity within the first 24–48 h of contact with fibroblast cells, whereas some samples exhibited lower cell viability (as low as 73%) after 24‐h, cell viability improved over time, exceeding 80% for all samples after 48 h. This initial drop in cell viability within 24 h was attributed to the interaction between nZnO and the cells, but subsequent growth and proliferation of the remaining cells led to increased viability. Consequently, the study concluded that the produced scaffold samples, including those with nZnO, were fully biocompatible and suitable for applications involving fibroblast cells [50].

Cell viability and proliferation are paramount factors in wound healing, as they reflect the scaffold′s ability to support and enhance cellular activity, ultimately contributing to tissue repair and regeneration (Reference [1]). In our study, the evaluation of these critical parameters using the MTT assay at both 24 and 48 h yielded noteworthy results. At the 24‐h mark, our findings revealed a statistically significant increase in cell viability and proliferation in the amnion group with nZnO when compared with the control group.

Our comprehensive assessment, including macroscopic examination and histological evaluation at various time points, shows the advanced tissue repair, neovascularization, and collagen organization observed within the treatment group, particularly on Day 21, are indicative of the scaffold′s contribution to enhanced wound closure. Selim et al. [41] conducted an in vitro and in vivo study on the efficacy of the biosynthesized form of nZnO in the burn wounds of rats with burn wound. Their study observed that nZnO significantly decreased the immunoreactivity of inflammatory markers such as IL‐6 and TNF‐*α* in the skin tissues of treated groups. These markers are typically elevated in response to tissue injury and infections, indicating that nZnO helps in mitigating inflammation. Their histopathological evaluations showed that the nZnO‐treated group exhibited low levels of fibrosis and mature surface epithelialization with keratinization. This suggests that nZnO not only aids in reducing inflammation but also facilitates the reepithelialization process, which is crucial for effective wound healing. Over the treatment period of 15 days, the wounds in the nZnO treated group showed significant improvement, with reduced size and signs of healing, such as hair regrowth and minimal scarring.

Regarding the limitations of the present study, future research should employ porcine or rabbit skin models, as these systems more closely replicate human dermal burn healing and may provide greater translational relevance. In addition, more comprehensive molecular investigations are required to elucidate the underlying mechanisms involved in the observed therapeutic effects. Finally, studies incorporating multiple dosing regimens may yield enhanced outcomes and offer deeper insight into dose–response relationships.

In conclusion, this study demonstrated that the novel biomaterial composed of nZnO integrated into hAM scaffolds shows immense potential for effectively treating second‐degree burn wounds. Our findings revealed accelerated reepithelialization, mature granulation tissue formation, and organized collagen deposition in rats treated with the hAM/nZnO scaffold compared with controls. Complete wound closure with no residual inflammation or infection was achieved within 21 days. Early intervention with this multifunctional biomaterial can help achieve rapid wound closure with minimal scarring, thereby improving clinical outcomes and quality of life for burn patients. The broad applicability of hAM and nZnO combinations suggests a promising future for this biomaterial in enhancing wound healing, tissue regeneration, and infection control across different clinical applications. There were some limitations to this study as well, we had a small sample size of 12 rats per group with limited long‐term follow‐up. Although initial histological assessments revealed promising early healing outcomes, a more comprehensive investigation correlating these findings with long‐term functional and cosmetic results is crucial. Additionally, comparisons with other nanoparticle‐based dressings could better characterize the efficacy of nZnO‐hAM scaffolds. Clinical studies establishing safety and efficacy in human burn patients are imperative before translation. Therefore, although our positive results highlight the potential of nZnO‐hAM composites for burn wound care, additional preclinical data followed by controlled human trials are warranted to further validate performance.

**Nomenclature**
hAM: human amniotic membrane
MDR: multidrug‐resistant
nZnO: zinc oxide nanoparticles
PBS: phosphate‐buffered saline
SEM: scanning electron microscopy
EDX: energy‐dispersive x‐ray
MTT: 3‐(4,5‐dimethylthiazol‐2‐yl)‐2,5‐diphenyltetrazolium bromide
DAPI: 4 ^′^,6‐diamidino‐2‐phenylindole
CO2: carbon dioxide
FACS: fluorescence‐activated cell sorter
ANOVA: analysis of variance
SPSS: Statistical Package for Social Sciences

## Author Contributions

All authors contributed to the article equally.

## Funding

No funding was received for this manuscript.

## Disclosure

The corresponding author (M.J.) affirms that this manuscript is an honest, accurate, and transparent account of the study being reported; that no important aspects of the study have been omitted; and that any discrepancies from the study as planned (registered as IR.SUMS.AEC.1401.129) have been explained. All authors approved the submitted version.

## Ethics Statement

The ethics committee of the Golestan University of Medical Sciences approved this study (Approval Number IR.SUMS.AEC.1401.129).

## Conflicts of Interest

The authors declare no conflicts interest.

## Data Availability

The datasets used and/or analyzed during the current study are available from the corresponding author upon reasonable request.
